# Ribosomal Biogenesis and Translational Flux Inhibition by the Selective Inhibitor of Nuclear Export (SINE) XPO1 Antagonist KPT-185

**DOI:** 10.1371/journal.pone.0137210

**Published:** 2015-09-04

**Authors:** Yoko Tabe, Kensuke Kojima, Shinichi Yamamoto, Kazumasa Sekihara, Hiromichi Matsushita, Richard Eric Davis, Zhiqiang Wang, Wencai Ma, Jo Ishizawa, Saiko Kazuno, Michael Kauffman, Sharon Shacham, Tsutomu Fujimura, Takashi Ueno, Takashi Miida, Michael Andreeff

**Affiliations:** 1 Section of Molecular Hematology and Therapy, Department of Leukemia, The University of Texas M. D. Anderson Cancer Center, Houston, TX, United States of America; 2 Department of Clinical Laboratory Medicine, Biomedical Research Center Graduate School of Medicine, Juntendo University, Tokyo, Japan; 3 Leading Center for the Development and Research of Cancer Medicine, Biomedical Research Center Graduate School of Medicine, Juntendo University, Tokyo, Japan; 4 Department of Laboratory Medicine, Tokai University of Medicine, Kanagawa, Japan; 5 Department of Lymphoma and Myeloma, The University of Texas M. D. Anderson Cancer Center, Houston, TX, United States of America; 6 Laboratory of Proteomics and Biomolecular Science, Biomedical Research Center Graduate School of Medicine, Juntendo University, Tokyo, Japan; 7 Karyopharm Therapeutics Inc., Natick, MA, United States of America; University of Illinois at Chicago, UNITED STATES

## Abstract

Mantle cell lymphoma (MCL) is an aggressive B-cell lymphoma characterized by the aberrant expression of several growth-regulating, oncogenic effectors. Exportin 1 (XPO1) mediates the nucleocytoplasmic transport of numerous molecules including oncogenic growth-regulating factors, RNAs, and ribosomal subunits. In MCL cells, the small molecule KPT-185 blocks XPO1 function and exerts anti-proliferative effects. In this study, we investigated the molecular mechanisms of this putative anti-tumor effect on MCL cells using cell growth/viability assays, immunoblotting, gene expression analysis, and absolute quantification proteomics. KPT-185 exhibited a p53-independent anti-lymphoma effect on MCL cells, by suppression of oncogenic mediators (e.g., XPO1, cyclin D1, c-Myc, PIM1, and Bcl-2 family members), repression of ribosomal biogenesis, and downregulation of translation/chaperone proteins (e.g., PIM2, EEF1A1, EEF2, and HSP70) that are part of the translational/transcriptional network regulated by heat shock factor 1. These results elucidate a novel mechanism in which ribosomal biogenesis appears to be a key component through which XPO1 contributes to tumor cell survival. Thus, we propose that the blockade of XPO1 could be a promising, novel strategy for the treatment of MCL and other malignancies overexpressing XPO1.

## Introduction

Mantle cell lymphoma (MCL) is an aggressive subtype of B-cell lymphoma and frequently resistant to standard chemotherapy [1]. MCL is characterized by the t(11,14)(q13;32) translocation that results in aberrant expression of cyclin D1 [2]. Although overexpressed cyclin D1 drives cell-cycle progression, causes instability in the G_1_-S checkpoint, and pronounced genetic instability, cyclin D1 overexpression itself is not sufficient for the development of MCL, suggesting that additional genetic events are necessary for development of this disease [3]. About 20% of MCL cases with increased nuclear pleomorphism are classified as blastoid MCL variants that have acquired additional genetic abnormalities such as mutated p53 [4]. Because of the multitude of signaling pathways that are dysregulated in MCL, a novel strategy aimed at restoring critical anti-oncogenetic pathways, especially targeting p53-independent signaling, is of considerable interest.

Nuclear-cytoplasmic transport of numerous molecules, including tumor suppressor and growth regulatory proteins, certain RNA species, and ribosomal subunits is mediated by the karyopherin family of proteins [5]. Exportin 1 (XPO1, also known as CRM1), is a major nuclear exporter of many tumor suppressor and growth regulatory proteins including p53, p73, Rb, p21, p27, Foxo, and NPM1 [6–8]. XPO1 can also be involved in the nuclear export of endogenous mRNAs including *cyclin D1* mRNA using adaptor proteins such as eukaryotic translation initiation factor 4E (eIF4e) in human cells [9]. Other important cargos of XPO1 are ribosomal subunits and RNAs. Elevated expression of XPO1 has been reported in the hematologic and solid tumors, and its overexpression is correlated with poor prognosis [10]. We have reported that the overexpression of XPO1 is associated with poor clinical outcomes in AML [11], and MCL [12].

Small-molecule selective inhibitors of nuclear export (SINE) that discriminately block XPO1-dependent nuclear export have been developed. SINEs specifically and irreversibly bind to the Cys528 residue in the cargo-binding groove of XPO1. Significant anti-leukemia activity of SINEs with negligible toxicity towards normal hematopoietic cells has been reported [10]. SINEs reportedly exhibit p53-dependent [11, 12] and -independent [13] anti-leukemia/lymphoma activities. However, the mechanisms of p53-independent apoptosis induced by SINEs have not been fully elucidated. In this study, we investigated the molecular anti-tumor mechanisms of the SINE KPT-185 in MCL cells. We report a critical function of XPO1 in ribosomal biogenesis, a key constituent of MCL cell survival, which suggest that XPO1 blockade by SINE compounds could be a promising, multi-targeted, and novel treatment strategy for MCL and other malignancies.

## Materials and Methods

### Cell Lines and Culture Conditions

The MCL cell lines Z138, JVM2, MINO, and Jeko-1 were used in this study [14]. The Z138 (blastoid-variant) and JVM2 cells have wt-*p53*, and the Jeko-1 and MINO cells harbor mutant *p53* [15]. The cells were cultured in RPMI 1640 containing 15% fetal bovine serum and 1% penicillin/streptomycin. In certain experiments, the cells were cultured with the indicated concentration of KPT-185 (provided by Karyopharm Therapeutics Inc., Natick, MA). JVM2 and Z138 cells were transduced with retroviruses encoding either p53-specific shRNA (nucleotides 611–629, Genbank NM000546) or scrambled shRNA and stable shRNA-expressing cells were generated [16].

### Cell Growth, Apoptosis, and Cell-Cycle Analysis

Cell viability was assessed by the Trypan blue dye exclusion method as described previously [17], and cell proliferation was determined by the CellTiter 96 AQueous One Solution Cell Proliferation Assay (MTS; Promega, Madison, WI) according to the company’s protocol. Apoptotic cell death was assessed by the annexin V–binding assay and cell-cycle distribution was analyzed by flow cytometric analysis of propidium iodine (PI)-stained nuclei as described previously [18].

### Immunoblot Analysis

Immunoblotting was performed as described previously [18]. The following antibodies were used: α-tubulin and β-actin (Sigma-Aldrich), p21^Cip1/WAF1^ and p27^KIP1^ (BD-Pharmingen, San Diego, CA); p53 (DO-7; Dako, Carpinteria, CA); BRCA1(Santa Cruz Biotechnology, Dallas, TX); Cdc2 (MBL, Nagoya, Japan); PIM-1 and XPO1 (marketed as anti-CRM1) and p-HSF1^Ser326^ (Abcam, Cambridge, MA); CDC25C, c-Myc, cyclin D1, Hsp70, 4E-BP1, phosphorylated- (p-) 4E-BP1^Thr37/Thr46^, p-Rb^Ser780^, S6 (S6K), p-S6 ribosomal protein (S6K)^Ser235/Ser236^, PUMA, HSF1, HSP70 and horseradish peroxidase–linked anti-mouse and anti-rabbit IgG (all from Cell Signaling Technology).

### iTRAQ Sample Labelling, Mass Spectrometry Analysis and Peptide Identifications

Isobaric tags for relative and absolute quantification (iTRAQ), a chemical labeling mass spectrometry method, has been performed following the manufacturer’s protocol (AB SCIEX, Framingham, MA, USA) [19, 20]. Protein identification and relative quantification were carried out using ProteinPilot Software Version 4.5 (AB SCIEX) [21]. Function definitions of the variable protein contents were searched against the Swissport database (Release, 10/16/2013). Protein ratios were normalized using the overall median ratio for all the peptides in the sample for each separate ratio in every individual experiment. A confidence cutoff for protein identification > 95% was applied. The specific pathway alteration was identified by Metacore (GeneGo, St. Joseph, MI) or KEGG ontology analysis (Kyoto University, Japan) [22].

### Gene Expression Analysis

Messenger RNA expression levels were quantified using TaqMan gene expression assays using TaqMan low-density array cards (TLDAs) (*PUMA*, Hs00248075_m1; *p21*, Hs00355782_m1; *GAPDH*, Hs99999905_m1, Applied Biosystems, Foster City, CA) or Reverse transcription real time quantitative PCR (RQ-PCR) (HSF1, Hs00232134_m1; GAPDH, Hs99999905_m1, Applied Biosystems) on a 7900HT Fast Real-Time PCR System. Relative quantification between different samples was determined according to the 2–ΔΔCt. Cells were treated by KPT-185 for 18 h (100 nM for Z138, MINO, and Jeko-1, 500 nM for JVM2).

### mRNA Hybridization and Gene-Expression Profiling

JVM2 cells transfected with control shRNA (shC JVM2) or p53-specific shRNA (shp53 JVM2) were left untreated or treated for 18 hr with 100 nM KPT-185, using 3 independent replicates for each shRNA and condition. Total RNA was extracted using the RNAqueous kit (Ambion, Austin, TX). After confirmation of RNA quality using a Bioanalyzer 2100 instrument (Agilent), 300 ng of total RNA was amplified and biotin-labeled through an Eberwine procedure using an Illumina TotalPrep RNA Amplification kit (Ambion) and hybridized to Illumina HT12 version 4 human whole-genome arrays. Processing of bead-level data was by methods previously described [23]. In brief, outlier-filtered bead values underwent model-based background correction [24], quantile normalization, filtering for probe quality [25], and log2 transformation. Candidate differentially-expressed probes (DEPs) were determined by comparing results for each KPT185-treated replicate to the average of corresponding untreated replicates. DEPs which changed in the same direction (up or down) after KPT-185 treatment in at least 5 of 6 comparisons (3 for shC and 3 for shp53) by at least an absolute log2 value of 0.5 were used for pathway analysis by Ingenuity Pathway Analysis (IPA) software (Ingenuity systems, Mountain View, CA). The array data has been deposited in the Gene Expression Omnibus (GEO) database. The GEO accession number for this data is GSE70479.

### Statistical Analyses

Statistical analyses were performed using a two-tailed Student’s *t*-test. Statistical significance was considered when the P-value was ≤ 0.05. Where indicated, the results are expressed as the mean ± standard deviation (SD) of triplicate samples.

## Results

### KPT-185 Exhibits Anti-Lymphoma Effect Both on p53 Wild-Type and Mutant MCL Cells

**Fig 1:**
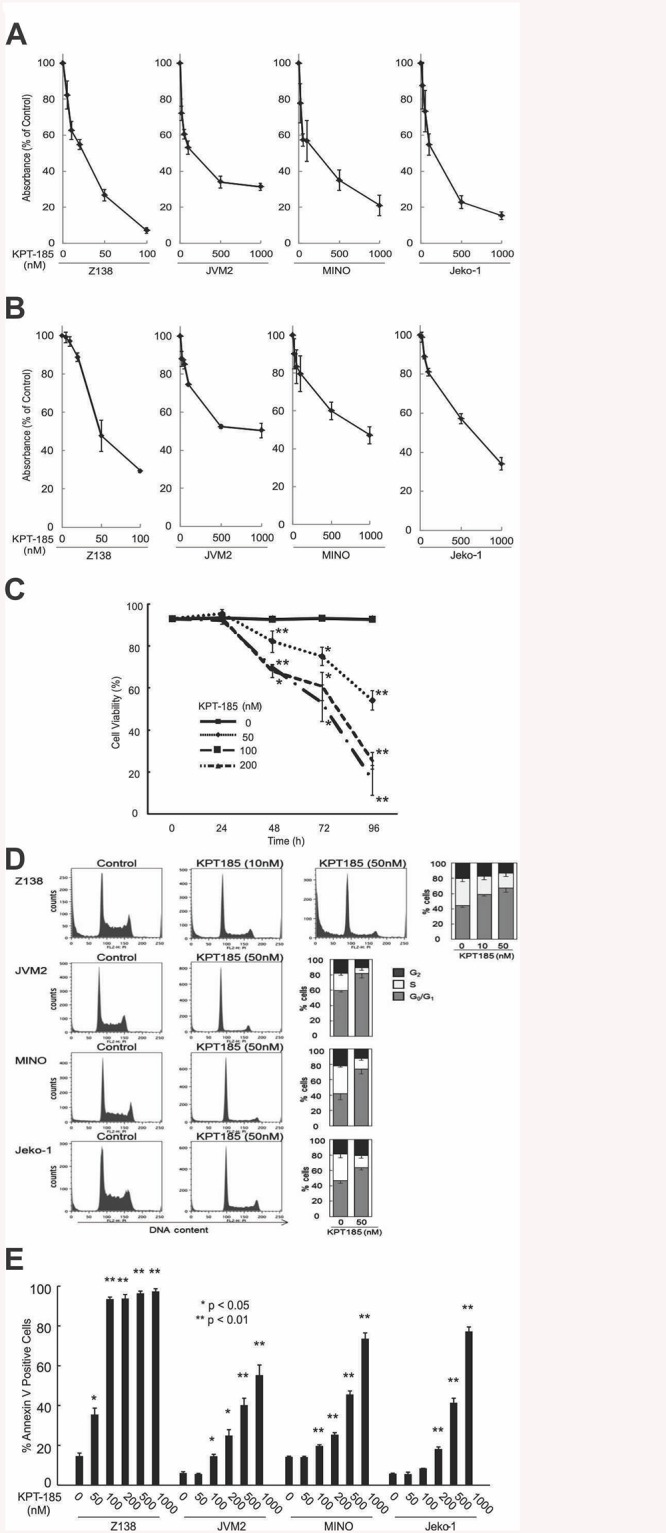
KPT-185 induced cell growth inhibition, apoptosis, and cell cycle arrest in MCL. Cells of wt-*p53* lines Z138 and JVM-2 or the mt-*p53* lines MINO and Jeko-1 were plated at a density of 2 x 10^5^ cells per mL and treated with the indicated concentrations of KPT-185. After 72 h, the effect on cell growth was assessed by the MTS test. Inhibition of cell growth is displayed as percent absorbance of untreated control cells. The concentrations of KPT-185 at which cell growth is inhibited by 50% (i.e., the IC50 concentration) was18 nM for Z138, 141 nM for JVM-2, 132 nM for MINO, and 144 nM for Jeko-1 (A). The percentage of dead cells was quantified by the tTypan blue dye exclusion method. The effective dose for cell killing of approximately 50% of the population (i.e., the ED50 concentration) after a 72-h exposure to KPT-185 was 57 nM for Z138 cells, 770 nM for JVM-2 cells, 917 nM for MINO cells, and 511 nM for Jeko-1 cells (B). Z138 cells were exposed to 50, 100, and 200nM KPT-185 for 24, 48, 72 and 96 h and assessed for cell growth as described in A. (C) The percentage of G_0_-G_1_, S, and G_2_-M phase cells in the viable cell population was assessed at 48 h by PI flow cytometry (histograms for representative samples are shown). Graphs show the mean ± SD of results of three independent experiments (D). The percentage of apoptotic MCL cells was quantified by annexin V/PI staining 72 h following the KPT-185 treatment as described above. The effective dose for cell killing of approximately 50% of the population (i.e., the ED50 dose) after a 72-h exposure to KPT-185 was 62 nM for Z138 cells, 910 nM for JVM-2 cells, 67 nM for MINO cells, and 618 nM for Jeko-1 cells (E). Graphs show the mean ± SD of results of three independent experiments. *p<0.05, **p<0.01.

We first examined the effect of KPT-185 on the proliferation of MCL cells with differing *p53* mutational status. Treatment with KPT-185 resulted in a dose- and time-dependent cell growth inhibition in all the MCL cells examined irrespective of their *p53* mutational status. The most prominent cell growth inhibition by KPT-185 was observed in the blastoid-variant Z138 cells (Fig 1A and 1C). Flow cytometric analysis of PI-stained cell nuclei showed that KPT-185 triggered a G_1_ phase accumulation of the cell cycle with concomitant decrease in the number of cells in S-phase cell population compared to controls in all tested cells (Fig 1D). Next, we determined that KPT-185 further exhibited a dose-dependent pro-apoptotic effect as evidenced by an increase in annexin V positivity in all of the MCL lines, with Z138 cells being extremely sensitive in this regard (Fig 1E).

**Fig 2:**
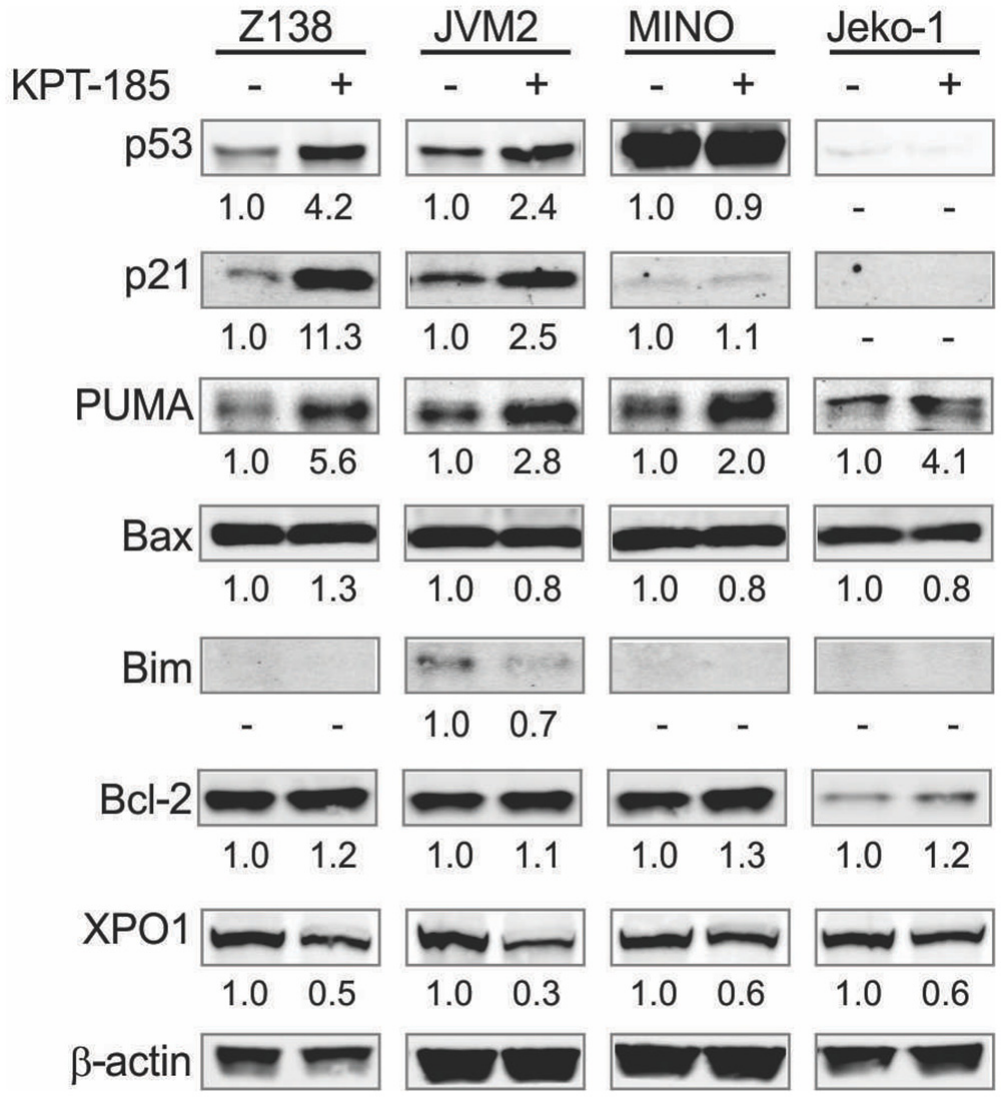
KPT-185 modulates XPO1 and Bcl-2 family members in MCL cells. After an 18-h treatment with KPT-185 (50 nM for Z138, 200 nM for JVM2, MINO and Jeko-1), cells were lysed and analyzed by immunoblot. The results are representative of three independent experiments, and the intensity, compared to that of β-actin, of the immunoblot signals was quantified using ImageJ software.

To assess whether p53 was activated by KPT-185 treatment, we examined protein expression levels of p53 and p53 target proteins p21 and PUMA. KPT-185 increased p53 expression in wt-*p53* bearing Z138 and JVM2 cells, followed by an increase in the classic p53 target p21. In mt-*p53* MINO and Jeko-1 cells no changes in p53 and p21 were detected following KPT-185 treatment. Of note, consistent increases in proapoptotic PUMA after KPT-185 treatment were evident in immunoblot analysis irrespective of p53 status (Fig 2). Concordant with these changes in protein expression, KPT-185 treatment upregulated the classical p53 target *p21* mRNA only in wt-*p53* bearing Z138 and JVM2 cells but not in mt-*p53* MINO and Jeko-1 cells (fold change; 2.2 for Z138, 3.1 for JVM-2, 1.2 for MINO, 1.3 for Jeko-1), and induced *PUMA* mRNA in all tested MCL cells irrespective of p53 status (fold change; 2.5 for Z138, 3.3 for JVM-2, 3.3 for MINO, 2.6 for Jeko-1) as assessed by PCR using TLDAs. These results suggest that KPT-185 induces p53-independent effects as well as p53 signaling activation in wt-p53 MCL cells. No significant change of pro-apoptotic Bim and Bax proteins or anti-apoptotic Bcl-2 protein was observed after KPT-185 treatment. KPT-185 decreased XPO1 in all tested MCL lines (Fig 2).

### KPT-185 Induces a Coordinated Downregulation of Proliferation-Related Genes in MCL

**Fig 3:**
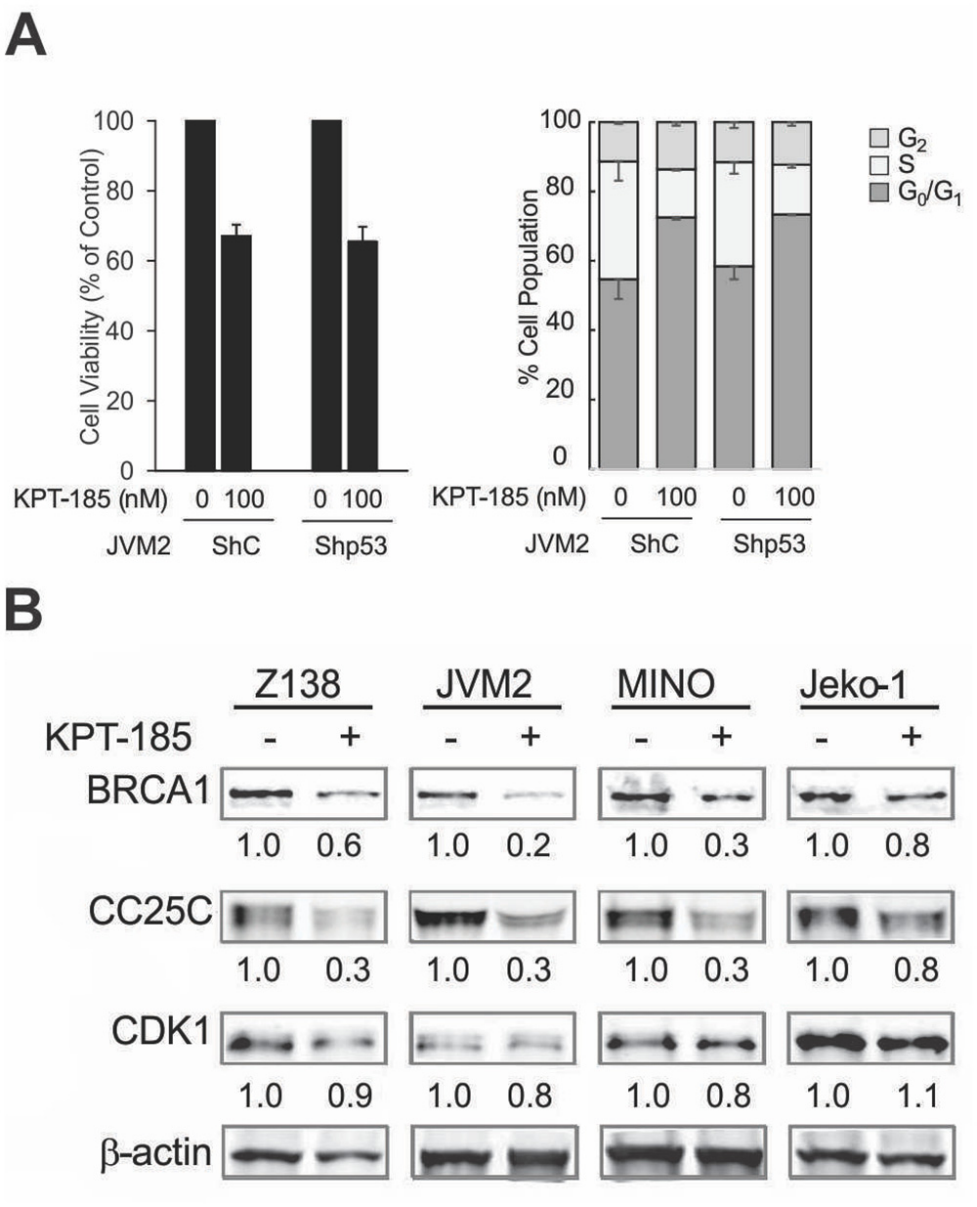
KPT-185 represses cell viability and cell cycle progression independent of p53-status. JVM2 cells stably transfected with control shRNA (shC) or *p53*-specific shRNA (shp53) were treated with 100 nM KPT-185. (A) The cell viability was assessed by the Trypan blue dye exclusion method and displayed as percent of untreated control cells at 72 h. The percentage of G_0_-G_1_, S, and G_2_-M phase cells in the viable cell population was assessed at 48 h by PI flow cytometry (histograms for representative samples are shown). Graphs show the mean ± SD of results of three independent experiments. (B) After an 18-h treatment of KPT-185, protein expression levels of CDC25C, BRCA1, CDK1 were analyzed by immunoblot as described in Fig 2. The results are representative of two independent experiments.

We then utilized wt-*p53* bearing MCL cells stably transfected with control shRNA (shC) or *p53*-specific shRNA (shp53) to evaluate p53-independent multi-targeted activities of KPT-185. p53-shRNA reduced p53 protein levels in JVM-2 and Z-138 cells by ≥ 80% as determined by immunoblot analysis [12]. As shown in Fig 3A, KPT-185 treatment induced cell growth inhibition with reduced cell viability and significant S-phase reduction in JVM2 cells irrespective of *p53* knockdown (p < 0.01), which was also observed in Z-138 cells transfected with shC or shp53 (% of S-phase cells of control / 20 nM KPT-185 treated; shC 32.7±2.2 / 27.5±2.4, p < 0.05, shp53 28.0±2.7 / 21.9±2.0, p < 0.05).

To assess p53-independent growth-regulatory pathways affected by XPO1 inhibition, we investigated the gene expression changes in shC JVM2 or shp53 JVM2 by KPT-185 treatment. Global gene expression changes associated with KPT-185 and the uniformly-changed genes that were altered regardless of functional *p53* status were detected as described in Materials and Methods. A total of 2461 gene probes were altered by KPT-185 treatment (100 nM for 18 hours) by at least 0.5 log2 (~ 40%) in at least 3 of 6 total replicates of shC and shp53 JVM2 cells. More uniform changes, affecting both shC and shp53 JVM2 cells similarly in at least 5 of 6 replicates, were found in 337 genes (i.e., 178 downregulated genes and 159 upregulated genes, Table A in S1 File and Table B in S1 File, respectively). Integrated Pathway Analysis showed that KPT-185 caused a coordinated downregulation of proliferation-related genes; most of the significantly enriched top 10 canonical pathways represented the downregulation of cell cycle progression by KPT-185 (Table 1). Supporting the microarray data, KPT-185 reduced protein expression levels of CDC25C, BRCA1, CDK1 detected by immunoblotting in most cases of four MCL cells regardless of p53 mutation status. (Fig 3B).

**Table 1:** Pathway analysis of genes in JVM-2 cells transfected with control shRNA or p53-specific shRNA consistently altered by KPT185.

Ingenuity Canonical Pathway	*p* value	ratio	Gene Name
Down-regulated			
Mitotic Roles of Polo-Like Kinase	< 0.0001	0.143	KIF23,CDC25C,PLK4,PTTG1,PRC1,CCNB2,FBXO5,PLK1,CDK1,KIF11
Cell Cycle: G2/M DNA Damage Checkpoint Regulation	< 0.0001	0.125	CDC25C,TOP2A,CCNB2,PLK1,BRCA1,CDK1
Role of CHK Proteins in Cell Cycle Checkpoint Control	< 0.0001	0.105	PCNA,CDC25C,PLK1,BRCA1,CDK1,RFC3
GADD45 Signaling	0.0003	0.136	PCNA,BRCA1,CDK1
DNA damage-induced 14-3-3σ Signaling	0.0003	0.143	CCNB2,BRCA1,CDK1
ATM Signaling	0.0006	0.066	CDC25C,CCNB2,BRCA1,CDK1
Pyridoxal 5'-phosphate Salvage Pathway	0.0014	0.055	NEK2,SGK1,PLK1,CDK1
dTMP De Novo Biosynthesis	0.0038	0.014	TYMS,NADPH
Salvage Pathways of Pyrimidine Ribonucleotides	0.0047	0.039	NEK2,SGK1,PLK1,CDK1
Up-regulated			
Germ Cell-Sertoli Cell Junction Signaling	0.0040	0.0305	TUBB3,JUP,ACTG2,GSN,TUBB2B

The significance of the association between the data set and the canonical pathway was determined based on a ratio of the number of genes from the data set that map to the pathway divided by the total number of genes that map to the canonical pathway and a *p*-value calculated using Fischer’s exact test determining the probability that the association between the genes in the data set and the canonical pathway is due to chance alone.

### KPT-185 Impairs Ribosome Biogenesis in MCL

To assess the protein(s) driving proliferation that are exported by KPT-185 and to identify the signaling pathways involved in this regulation, iTRAQ proteomics was employed for exhaustive protein expression analysis. Because XPO1 has multiple cargos including p53, and the clonal heterogeneity of MCL might reflect the functional differences [2], we utilized the different cell lines bearing wt-p53 Z138 and mt-p53 Jeko-1 cells. Since the gene expression analysis detected that XPO1 inhibition by KPT-185 induced a coordinated downregulation of proliferation-related genes and inhibition of cell cycle progression pathways, we used KPT-185 concentrations near to IC50 (50nM for Z138 and 100nM for Jeko-1) to detect the similarly affected proteins for cell growth inhibition of these cells by KPT-185.

**Fig 4:**
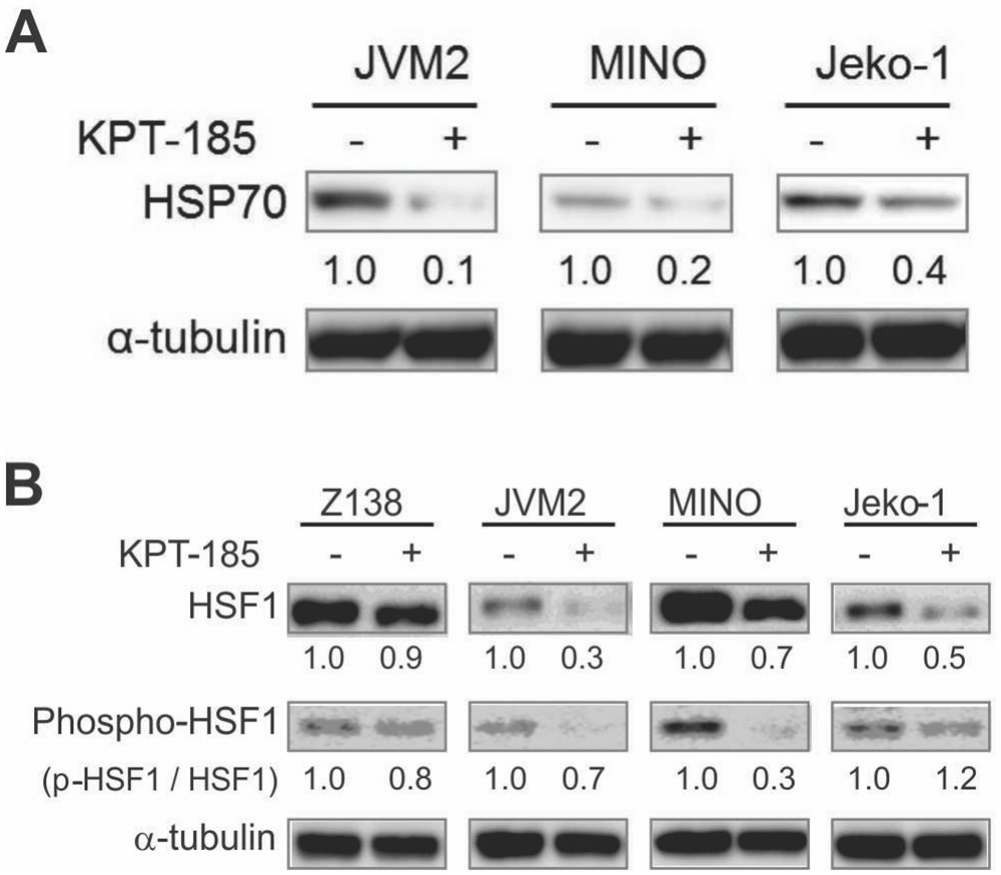
KPT-185 diminishes HSP70, HSF1 and p-HSF1^Ser326^ expression. After an 18-h treatment of KPT-185 (i.e., 50 nM for Z138, 100 nM for JVM2, MINO, and Jeko-1), the cells were lysed and analyzed HSP70 (A) and HSF1 and p-HSF1Ser326 expression (B) by immunoblot as described in Fig 2. α-tubulin was used as a loading control. The intensity, compared to that of α-tubulin or p-HSF1 / HSF1 levels after background subtraction were obtained using ImageJ software. The results are representative of three independent experiments.α.

In Z138 and Jeko-1 cells, a total of 2252 and 2176 unique proteins were identified including 137 and 112 proteins significantly altered by KPT-185 treatment, respectively. As shown in Table 2, 74 proteins consistently altered (62 downregulated and 12 upregulated) by KPT-185 and 81% of the downregulated proteins (i.e., 50 of 62) were ribosomal proteins, suggesting that KPT-185 strongly inhibited ribosomal biogenesis. iTRAQ further detected the significant and consistent repression of translation initiation and elongation factors such as eukaryotic translation initiation factor 4A1 (EIF4A1/PIM2), eukaryotic translation elongation factor 1-alpha 1 (EEF1A1), and eukaryotic elongation factor 2 (EEF2) after KPT185 treatment in both tested cell lines. The chaperone proteins heat shock protein 70 (HSP70) and phospho-heat shock protein 90 (phospho-HSP90) were also downregulated by KPT-185. The downregulation of HSP70 by KPT-185 was confirmed by immunoblotting (Fig 4A).

**Table 2:** Consistent changes in proteins in Z138 and Jeko-1 cells detected by iTRAQ; after KPT185 treatment.

Protein	Gene name	Z138		Jeko-1	
		p-value*	KPT-185/control	p-value*	KPT-185/control
			ratio		ratio
down-regulated proteins					
Large ribosomal subunit (60S) proteins					
60S ribosomal protein L10	RPL10	0.0007	0.7574	0.0001	0.7622
60S ribosomal protein L10a	RPL10A	0.0034	0.8146	0.0003	0.8046
60S ribosomal protein L11	RPL11	0.04	0.8132	0.018	0.7992
60S ribosomal protein L12	RPL12	0.0335	0.8685	0.0169	0.8222
60S ribosomal protein L13	RPL13	0.0004	0.799	0.0012	0.7847
60S ribosomal protein L13a	RPL13A	0.0141	0.8387	0.0008	0.7947
60S ribosomal protein L14	RPL14	0.0061	0.791	0.0129	0.7474
60S ribosomal protein L15	RPL15	0.002	0.8255	0.0003	0.8036
60S ribosomal protein L17	RPL17	0.0202	0.8305	0.0118	0.7821
60S ribosomal protein L18	RPL18	0.0082	0.7628	0.0057	0.7437
60S ribosomal protein L18a	RPL18A	0.0071	0.8195	0.0076	0.8278
60S ribosomal protein L19	RPL19	0.0235	0.8478	0.0056	0.8094
60S ribosomal protein L23	RPL23	0.0256	0.8061	0.009	0.8155
60S ribosomal protein L23a	RPL23A	0.0025	0.8117	0.001	0.779
60S ribosomal protein L24	RPL24	0.0074	0.7566	0.0156	0.7327
60S ribosomal protein L27	RPL27	0.0075	0.8104	0.0014	0.776
60S ribosomal protein L27a	RPL27A	0.0087	0.7668	0.0064	0.7201
60S ribosomal protein L28	RPL28	0.0052	0.8261	0.0013	0.7831
60S ribosomal protein L3	RPL3	0	0.812	0	0.8052
60S ribosomal protein L30	RPL30	0.0315	0.8432	0.0049	0.819
60S ribosomal protein L34	RPL34	0.0243	0.8201	0.0088	0.7775
60S ribosomal protein L35a	RPL35A	0.0468	0.8577	0.0038	0.8166
60S ribosomal protein L36	RPL36	0.0305	0.7836	0.0121	0.723
60S ribosomal protein L37	RPL37	0.0424	0.7846	0.0449	0.7224
60S ribosomal protein L4	RPL4	0	0.7754	0	0.7608
60S ribosomal protein L5	RPL5	0.0257	0.8921	0.0079	0.8704
60S ribosomal protein L6	RPL6	0	0.7901	0	0.7785
60S ribosomal protein L7	RPL7	0.0023	0.8445	0.0014	0.8334
60S ribosomal protein L7a	RPL7A	0.0003	0.809	0.0008	0.8101
60S ribosomal protein L8	RPL8	0.0488	0.8193	0.0343	0.7778
60S ribosomal protein L9	RPL9	0.0061	0.8167	0.0004	0.7867
60S acidic ribosomal protein P0	RPLP0	0.0013	0.8371	0.0021	0.8459
Small ribosomal subunit (40S) proteins					
40S ribosomal protein S10	RPS10	0.0009	0.784	0.0014	0.7786
40S ribosomal protein S11	RPS11	0.0017	0.8143	0.0003	0.7564
40S ribosomal protein S13	RPS13	0.0333	0.8666	0.0069	0.8311
40S ribosomal protein S15a	RPS15A	0.0087	0.7287	0.0017	0.6979
40S ribosomal protein S16	RPS16	0.0016	0.8143	0.0002	0.7658
40S ribosomal protein S17-like	RPS17L	0.0328	0.8531	0.0147	0.8268
40S ribosomal protein S18	RPS18	0.0045	0.8213	0.0001	0.7372
40S ribosomal protein S19	RPS19	0.0013	0.8189	0	0.7414
40S ribosomal protein S2	RPS2	0.0121	0.849	0.0025	0.7823
40S ribosomal protein S3	RPS3	0	0.8151	0	0.7585
40S ribosomal protein S3a	RPS3A	0.0005	0.8366	0	0.7894
40S ribosomal protein S4, X isoform	RPS4X	0.0019	0.8003	0.0001	0.8184
40S ribosomal protein S5	RPS5	0.0199	0.8357	0.0035	0.7664
40S ribosomal protein S6	RPS6	0.0091	0.8	0.0036	0.7767
40S ribosomal protein S7	RPS7	0.0335	0.788	0.0014	0.7812
40S ribosomal protein S8	RPS8	0.0028	0.8234	0.0005	0.7745
40S ribosomal protein S9	RPS9	0.0001	0.8019	0	0.752
40S ribosomal protein SA	RPSA	0.0046	0.8591	0.0095	0.8411
Elongation factor 2	EEF2	0.0001	0.9027	0.0003	0.9163
Eukaryotic initiation factor 4A-I (PIM2)	EIF4A1	0.0023	0.8667	0.0155	0.873
Importin subunit alpha-2	KPNA2	0.0006	0.6583	0.0047	0.8308
Exportin-1	XPO1	0.0014	0.723	0.0001	0.7854
Ribonucleoside-diphosphate reductase subunit M2	RRM2	0.0014	0.5415	0.0013	0.6767
ATP-binding cassette sub-family F member 2	ABCF2	0.0409	0.8459	0.001	0.7975
Heat shock cognate 71 kDa protein	HSPA8	0.0003	0.8067	0.0003	0.8829
Fatty acid synthase	FASN	0.0013	0.9129	0.0005	0.9131
Guanine nucleotide-binding protein subunit beta-2-like 1	GNB2L1	0.0004	0.8386	0	0.7956
Insulin-like growth factor 2 mRNA-binding protein 1	IGF2BP1	0.0274	0.8886	0.0165	0.8707
Polyadenylate-binding protein 1	PABPC1	0.0034	0.8354	0.0007	0.857
Splicing factor, proline- and glutamine-rich	SFPQ	0.0058	0.8675	0.011	0.8801
up-regulated proteins					
Histone H2A type 1-A	HIST1H2AA	0.0027	3.979	0.0231	1.5336
60 kDa heat shock protein, mitochondrial	HSPD1	0.0327	1.0685	0.0254	1.0664
Prohibitin	PHB	0.0004	1.2134	0.0001	1.2541
Prohibitin-2	PHB2	0.0002	1.2149	0.0002	1.2144
6-phosphofructokinase, muscle type	PFKM	0.0242	1.1211	0.0231	1.2271
Phosphoglycerate kinase 1	PGK1	0.0055	1.0995	0.0259	1.078
Isoform B of Phosphate carrier protein, mitochondrial	SLC25A3	0.0055	1.2836	0.0012	1.2593
ATP synthase subunit alpha, mitochondrial	ATP5A1	0.0019	1.1216	0.0083	1.1039
Hepatoma-derived growth factor	HDGF	0.0344	1.1157	0.0364	1.1194
Leukotriene A-4 hydrolase	LTA4H	0.0463	1.1572	0.011	1.2134
Malate dehydrogenase, cytoplasmic	MDH1	0.0064	1.134	0.0173	1.1014
Malate dehydrogenase, mitochondrial	MDH2	0.0003	1.1757	0.0091	1.1151

Z-138 and Jeko-1 cells were treated with KPT-185 (50 nM for Z138; 100 nM for JeKo-1) for 18 hours, and relative changes in protein levels were determined by using iTRAQ (isobaric tags for relative and absolute quantification).

Interestingly, all of these factors are targets of the multifaceted transcription factor heat shock factor 1 (HSF1), which has been reported to be a central transducer linking translational activity of ribosomal biogenesis and transcriptional regulation [26]. We therefore determined whether KPT-185 affected the expression levels of the HSF1 gene and/or protein. Real-time RT-PCR analysis showed no significant difference in the levels of HSF1 mRNA by KPT-185 treatment both in Z-138 and Jeko-1 cells (data not shown), suggesting that XPO1 inhibition did not affect HSF1 transcription. However, KPT-185 treatment strikingly downregulated HSF1 protein levels, accompanied by the concomitant suppression of HSF1 Ser326 phosphorylation in 3 of 4 tested MCL cell lines (Fig 4B). These data indicate that XPO1 positively regulates HSF1 via translational modulation, and that this process can be blocked by the inhibition of XPO1.

Among the 13 proteins that were consistently upregulated by KPT-185, the glucose metabolic kinases, ATP synthase and apoptosis related proteins such as histone H2, heat shock protein 60 (HSP60), and prohibitin were included. Along with down-regulation of ribosomal biogenesis, Metacore and KEGG GO analysis showed a consistent pathway alteration in Z138 and Jeko-1 cells after KPT185 treatment including down-regulation of translation initiation and up-regulation of glycolysis, gluconeogenesis and pyruvate metabolism (Table 3). Concordant with the immunoblot results (Fig 2), iTRAQ analysis detected XPO1 downregulation by KPT-185 in both Z138 and Jeko-1 cells (Table 2).

**Table 3:** Consistent pathway alteration in Z138 and Jeko-1 cells after KPT185 treatment.

Pathway type accession #	name	gene name	Z138			Jeko-1		
			number of peptides found	p-value*	KPT-185/control ratio	number of peptides found	p-value*	KPT-185/control ratio
Translation _Regulation of translation initiation				5.361E-08			7.009E-14	
P63244	Guanine nucleotide-binding protein subunit beta-2-like 1	GNB2L1	36	0.0004	0.8386	39	0	0.7956
P60842	Eukaryotic initiation factor 4A-I	EIF4A1	36	0.0023	0.8667	43	0.0155	0.873
P11940	Polyadenylate-binding protein 1	PABPC1	45	0.0034	0.8354	42	0.0007	0.857
P62753	40S ribosomal protein S6	RPS6	14	0.0091	0.8	11	0.0036	0.7767
Q14152	Eukaryotic translation initiation factor 3 subunit A	EIF3A				58	0.0112	0.9342
P41091	Eukaryotic translation initiation factor 2 subunit 3	EIF2S3				21	0.0066	0.8737
Glycolysis and gluconeogenesis				1.845E-03			1.185E-08	
P40925	Malate dehydrogenase, cytoplasmic	MDH1	19	0.0064	1.134	19	0.0173	1.1014
P40926	Malate dehydrogenase, mitochondrial	MDH2	41	0.0003	1.1757	32	0.0091	1.1151
P00558	Phosphoglycerate kinase 1	PGK1	49	0.0055	1.0995	46	0.0259	1.078
P08237	6-phosphofructokinase, muscle type	PFKM	23	0.0242	1.1211	12	0.0231	1.2271
P06733	Alpha-enolase	ENO1				80	0.0027	1.0986
P60174	Triosephosphate isomerase	TPI1				37	0.0328	1.0888
P04406	Glyceraldehyde-3-phosphate dehydrogenase	GAPDH				125	0.0343	1.0794
Pyruvate metabolism				6.262E-04			4.005E-04	
P40925	Malate dehydrogenase, cytoplasmic	MDH1	19	0.0064	1.134	19	0.0173	1.1014
P40926	Malate dehydrogenase, mitochondrial	MDH2	41	0.0003	1.1757	32	0.0091	1.1151
P09622	Dihydrolipoyl dehydrogenase	DLD	17	0.017	1.1428			
P23368	NAD-dependent malic enzyme, mitochondrial	ME2	16	0.0033	1.3219			
Q04760	Lactoylglutathione lyase	GLO1				18	0.032	1.131
P07195	L-lactate dehydrogenase B chain	LDHB				40	0.0034	1.1293
Transcription_Role of Akt in hypoxia induced HIF1 activation				1.241E-03			7.801E-04	
P11142	Heat shock cognate 71 kDa protein	HSPA8	102	0.0003	0.8067	85	0.0003	0.8829
P78371	T-complex protein 1 subunit	TCP1	49	0.0227	0.933			
P00558	Phosphoglycerate kinase 1	PGK1	49	0.0055	1.0995	46	0.0259	1.078
P06733	Alpha-enolase	ENO1				80	0.0027	1.0986

Protein accession numbers refer to SWISS-PROT or TrEMBL entries. Confidence score (a percentage measure of the confidence of the protein identification) for all proteins in the was 99%. All proteins are significantly different (p < 0.05) between control and KPT-185 treated cells.

*Individual p-values have been corrected for multiple comparisons. Expression changes of each of 3 independent experiments, comparing KPT-185 treated cells to untreated cells in JVM2 transfected with control shRNA (shC JVM2) or p53-specific shRNA (shp53 JVM2).

### KPT-185 Downregulates Cyclin D1, c-Myc, and PIM1 Expression and Inhibits mTOR Signaling in MCL Cells

**Fig 5:**
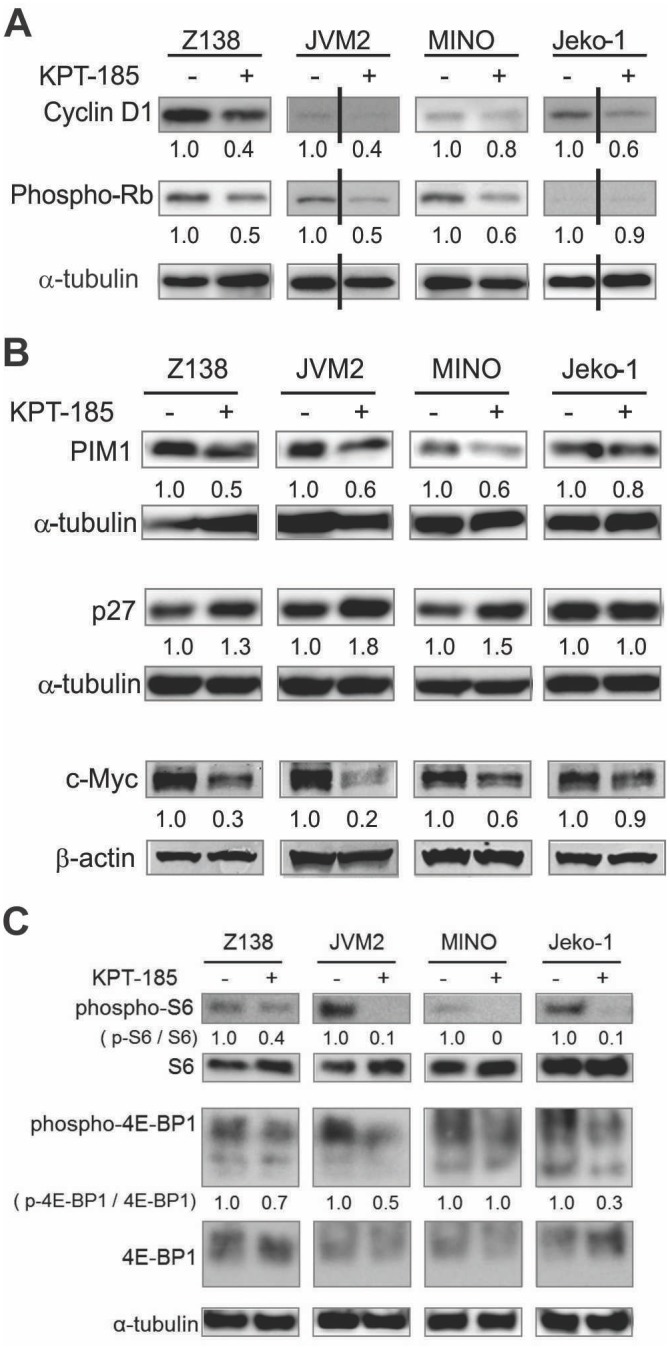
KPT-185 targets multiple signaling pathways in MCL cells. (A) After an 18-h treatment of KPT-185 (i.e., 50nM for Z138 and MINO, 100 nM for JVM2 and Jeko-1), Cyclin D1 and its downstream target phosphorylated Rb expression were analyzed by immunoblot. (B, C) After an 18-h treatment of KPT-185 (i.e., 50nM for Z138 and 100 nM for JVM2, MINO, and Jeko-1), PIM1 and p27KIP (B), phospho-S6, and phospho-4EBP1 (C) were analyzed by immunoblot. For c-Myc expression analysis, cells were treated with 500 nM KPT-185. α-tubulin was used as a loading control. The intensity, compared to that of α-tubulin or p-S6K / S6K, p-4E-BP1 / 4E-BP1 levels after background subtraction were obtained using ImageJ software. The results are representative of three independent experiments.

It has been shown that overexpression of XPO1 overcomes p16INK4a mediated checkpoint control [27]. Overexpression of cyclin D1, downstream of p16INK4a [28], is implicated in the pathogenesis of MCL, and XPO1 is known to modulate the nuclear export of cyclin D1 mRNA via adapter protein eukaryotic translation initiation factor 4E (eIF4E) [29]. We therefore investigated whether KPT-185 treatment affected cyclin D1 expression. Indeed, we observed downregulation of cyclin D1, which was accompanied by a substantial decrease of its target protein phospho-Rb after KPT-185 treatment (Fig 5A). Of note, blastoid-variant Z138 cells, highly sensitive to KPT185, showed significantly higher cyclin D1 baseline expression compared to other MCL cell lines.

Although cyclin D1 could be responsible for the anti-tumor effect of XPO1 inhibition, it is known that the overexpression of cyclin D1 itself is not sufficient for development of MCL [3]. Recently, it has been shown that *c-Myc* and *PIM1* mRNAs use XPO1 and the adapter protein eIF4e for their transport into the cytoplasm, which facilitates their translation [9, 30]. Overexpression of the oncogenic transcription factor c-Myc has been reported to be significantly associated with shorter overall survival in MCL [31], and collaboration of PIM1 with c-Myc is a critical mechanism defining cell cycle progression and tumorigenesis [32]. Immunoblot analysis detected KPT-185 induced downregulation of c-Myc and PIM1 and increase of p27^KIP^, a cyclin dependent kinase (CDK) inhibitor in all tested MCL cell lines except Jeko-1 which showed only minimal changes (Fig 5B), suggesting that XPO1 inhibition by KPT-185 may affect oncogenic c-Myc and PIM1 as well as cyclin D1 functions to different degrees in MCL cells. KPT-185 further downregulated phosphorylation levels of the mTOR substrates ribosomal protein S6 kinase (S6K) and/or eukaryotic translation initiation factor 4E (eIF4E)-binding protein 1 (4E-BP1) in most cases of tested MCL cells (Fig 5C).

## Discussion

**Fig 6:**
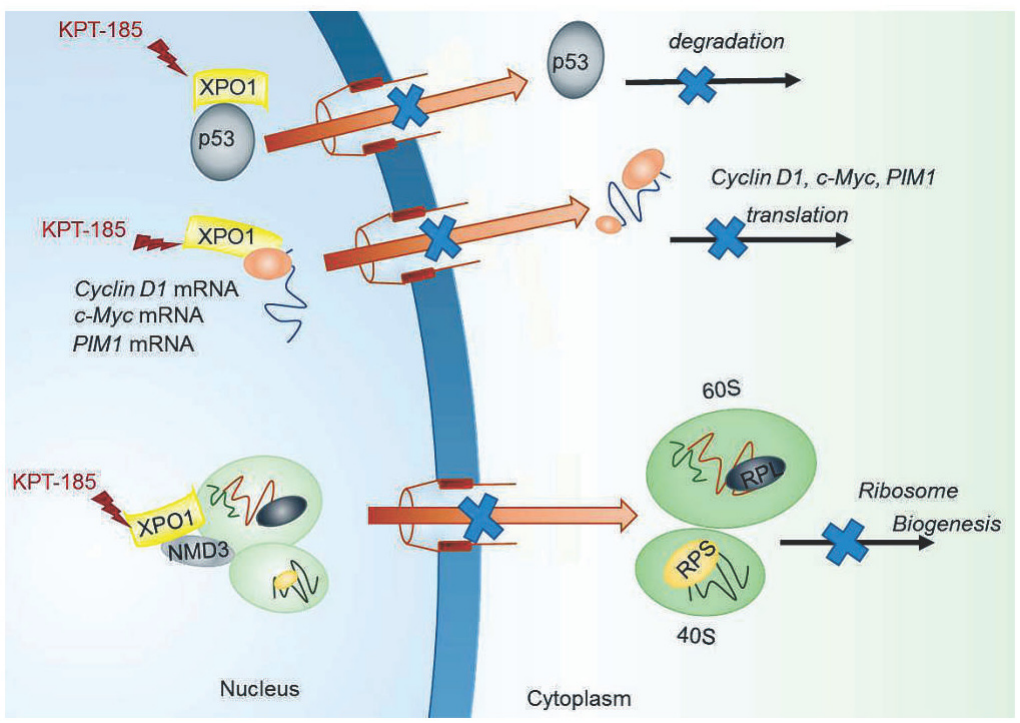
XPO1 inhibition by KPT-185 in MCL. XPO1 inhibition by KPT-185 impairs ribosomal biogenesis, in addition to blocking p53 degradation and inhibiting CyclinD1, c-Myc, and PIM1 translation in MCL. Please refer to the discussion for further details.

Ribosomal synthesis is a highly ordered process, and the ribosome functions as a central information hub in cancer cells [26]. We demonstrated that XPO1 inhibition by KPT-185 exhibited single-agent anti-proliferative activities against MCL cells via inhibition of multiple factors: ribosomal biogenesis and protein synthesis, the transcription factor HSF1, and the nuclear export of oncogenic mRNAs, including *cyclin D1*, *c-Myc* and *PIM1* (Fig 6). XPO1 mediates export of ribosomal subunits from the nucleus utilizing the nucleocytoplasmic shuttling adaptor protein NMD3 [33], and the inhibition of ribosomal biogenesis has been shown to impair DNA occupancy of HSF1 which regulates genes controlling heat-shock proteins, protein synthesis [34], and energy metabolism, important to tumor cell survival and proliferation [26]. We detected that KPT-185 induced reductions of total- and phosphoactivated-HSF1 along with its targets PlM2, HSP70, phospho-HSP90 and EEF1A1; the absence of effects on *HSF1* mRNA levels indicated that XPO1 inhibition repressed HSF1 translation but not transcription, through mechanisms that remain to be elucidated in MCL cells.

Although p53 has been recognized as a key player linking ribosomal biogenesis and cell-cycle repression [35], p53-independent impairment of ribosomal biogenesis via PIM and c-Myc downregulation has also been reported [36, 37]. We detected KPT-185-induced downregulation of PIM1 and c-Myc, whose mRNAs are known XPO1 cargos [9, 30]. XPO1 binds to *c-Myc* and *PIM1* mRNAs as well as *cyclin D1* mRNA via an adapter protein eukaryotic translation initiation factor 4E (eIF4e) [9, 30]. Interestingly, we found that KPT-185 decreased phosphorylated 4E-BP1, which allows 4E-BP1 binding to eIF4e and inhibition of eIF4e effects [38]. On the other hand, PIM1 kinase is known to interact with the ribosomal protein RPS19, one of the KPT-185 targeted ribosomal proteins in MCL cells (Table 2), and depletion of RPS19 causes proteasomal degradation of PIM1 [39] and p27^KIP^ stabilization, thus causing a block in cell-cycle progression regardless of p53 status [39]. iTRAQ proteomics showed that KPT-185 downregulated PIM2 irrespective of p53 status. PIM2 is known to be associated with an aggressive clinical course in B-cell lymphomas [40], and is involved in the regulation of mTOR complex 1 (mTORC1). Our immunoblot data showed that KPT-185 downregulated mTORC1 downstream targets, phospho-S6K and/or phospho-4E-BP1. Gene expression profiling further showed p53-independent downregulation by KPT-185 of several factors closely associated with PIM, c-Myc, and mTOR signaling, such as cell division cycle25C (CDC25C) [41], the global transcription factor pituitary tumor-transforming gene-1 (PTTG1) [42], and the mTORC1 substrate serum/glucocorticoid-regulated kinase 1 (SGK1) [43]. The p53 status-independent transcriptional induction of PUMA by KPT-185 indicates the role of additional transcriptional factors, such as an XPO1 cargo FOXO3a that is responsible for the upregulation of PUMA [44], or NF-κB, whose blockade by KPT-SINEs induced p53-independent depletion of MCL cells [13, 45]. Of note, KPT-185 strikingly targeted cyclin D1 and its downstream signaling in MCL cells, and the blastoid-variant Z138 with high baseline expression of cyclin D1 was the most sensitive to KTP-185 among the tested MCL cell lines, suggesting that cyclin D1 is a critical target of KPT-185 for its anti-tumor activity.

KPT-185 decreased XPO1 in all tested MCL cells, which is concordant with the previous reports of MCL and lung cancer cells [13, 46], in which KPT-185 induced proteasomal degradation of XPO1 protein despite its normally long half-life (24 hours) [46]. In our system, KPT-185 caused the biggest decline of XPO1 protein expression in JVM2 cells, which also showed the lowest sensitivity to KPT-185. Considering that SINEs irreversibly bind to the groove of XPO1 protein, which results in blockade of XPO1-directed protein export [10], the eventual degradation of XPO1 at longer time points might not directly affect the SINE XPO1 inhibition effects by KPT-185.

Unexpectedly, pathway analysis also detected the significant upregulation of glycolysis and gluconeogenesis in KPT-185 treated MCL cells. The nuclear localization of the transcription factor carbohydrate responsive element binding protein (ChREBP) is required for glucose metabolism [47, 48], and XPO1-associated nuclear export is involved in its inactivation [49]. Accumulation of ChREBP in the nucleus by KPT-185 might result in activation of aerobic glycolysis [47, 48], which plays an important role in sustaining tumor growth [50]. Further understanding of factors responsible for XPO-1 inhibition-induced anabolic metabolism may allow us to develop combination strategies with XPO-1 inhibitors.

The first specific nuclear export inhibitor Leptomycin B (LMB) [51] has been noted for off-target binding to proteins other than XPO1, contributing to toxicities [52]. Tai et al. [53] demonstrated that SINEs including KPT-185 blocked XPO1 with effects similar to shRNA knockdown of XPO1 in multiple myeloma, indicating that specific XPO1 inhibition by KPT-185 mediated anti-tumor properties, rather than an off-target effect. In this study, however, the off-target effects of KPT-185 have not been exhaustively studied and we cannot rule out contributions of XPO1-independent multi-targeted activities of KPT-185 to the observed phenomena in MCL cells.

The clonal heterogeneity of MCL might reflect the functional heterogeneity and complex pathogenesis of the disease [2]. Recently, the existence of multiple subclones in more than 50% of MCL cases has been reported [37]. The inhibition of ribosomal biogenesis by the depletion of pre-rRNA processor pescadillo nucleolar protein [54] caused the stabilization of p53, which led to cell cycle arrest in wt-p53 cells along with decreased expression of cyclin D1 and pRB phosphorylation/up-regulation of p27 [55]. At the same time, the functional, p53-independent anti-tumor mechanisms of ribosomal stress possibly reflect the process of neoplastic transformation, and, as such, could identify new targets for therapeutic applications [56]. Indeed, we demonstrated that increased XPO1 expression was associated with poor prognosis in MCL patients [12], suggesting that SINE/XPO1 antagonism by KPT-185 could be a promising strategy for the therapy of MCL.

An orally bioavailable SINE Selinexor (KPT-330) is the only NEI currently in Phase I/II human clinical trials in hematological and solid cancers [57], and has rapid absorption and dose-proportional pharmacokinetics with no accumulation [10, 53, 58]. Our findings are consistent with the first clinical results demonstrating complete responses in patients with leukemias and lymphomas [59, 60]. The inhibition of ribosomal biogenesis may also account for the observed common toxicities, in particular anorexia and weight loss, in AML patients treated with KPT-330 [60]. Consequently, the blockade of XPO1 by KPT-185, a SINE identified as a potent inhibitor of ribosomal biogenesis, is a novel and potentially promising strategy for the treatment of MCL, and possibly other XPO1-overexpressing tumors.

## Supporting Information

S1 File Lists of consistently down- (Table A in S1 File) or up-regulated (Table B in S1 File) genes by KPT-185.treatment in JVM-2 cells transfected with control shRNA or p53-specific shRNA. Mean fold-change was determined from the average of gene expression changes of each of 3 independent experiments in JVM2 transfected with control shRNA (shC JVM2) or p53-specific shRNA (shp53 JVM2) comparing KPT-185-treated cells to untreated controls.
(DOCX)
