# Supplementary material for: Ribosomal Biogenesis and Translational Flux Inhibition by the Selective Inhibitor of Nuclear Export (SINE) XPO1 Antagonist KPT-185
Source: PLoS One. 2015 Sep 4;10(9):e0137210. doi: 10.1371/journal.pone.0137210 (PMC4560410; doi:10.1371/journal.pone.0137210)
Supplement: S1 File — Mean fold-change was determined from the average of gene expression changes of each of 3 independent experiments in JVM2 transfected with control shRNA (shC JVM2) or p53-specific shRNA (shp53 JVM2) comparing KPT-185-treated cells to untreated controls. (DOCX) [file pone.0137210.s001.docx]

# **S1 File: Supplementary Information**

Table A. List of consistently downregulated genes by KPT-185 treatment in JVM-2 cells transfected with control shRNA or p53-specific shRNA.

Mean fold-change was determined from the average of gene expression changes of each of 3 independent experiments in JVM2 transfected with control shRNA (shC JVM2) or p53-specific shRNA (shp53 JVM2) comparing KPT-185-treated cells to untreated controls.

Table B. List of consistently upregulated genes by KPT-185treatment in JVM-2 cells transfected with control shRNA or p53-specific shRNA.

Mean fold-change was determined from the average of gene expression changes of each of 3 independent experiments in JVM2 transfected with control shRNA (shC JVM2) or p53-specific shRNA (shp53 JVM2) comparing KPT-185-treated cells to untreated controls.
